# Supplementary material for: Myocardial Notch1-Rbpj deletion does not affect NOTCH signaling, heart development or function
Source: PLoS One. 2018 Dec 31;13(12):e0203100. doi: 10.1371/journal.pone.0203100 (PMC6312338; doi:10.1371/journal.pone.0203100)
Supplement: S1 Table — (PDF) [file pone.0203100.s001.pdf]

Control embryo #1	RV		LV	
	CM thickness (µm)	TM thickness (µm)	CM thickness (µm)	TM thickness (µm)
Section #1	83,3	101	137	140
Section #2	78,9	88,8	155	129
Section #3	63,3	110	141	148
Mean	75,16666667	99,93333333	144,3333333	139

Control embryo #2	RV		LV	
	CM thickness (µm)	TM thickness (µm)	CM thickness (µm)	TM thickness (µm)
Section #1	101	72,1	161	161
Section #2	101	73,3	153	151
Section #3	92,5	111	150	157
Mean	98,16666667	85,46666667	154,6666667	156,3333333

Control embryo #3	RV		LV	
	CM thickness (µm)	TM thickness (µm)	CM thickness (µm)	TM thickness (µm)
Section #1	110.750	107.242	128.099	87.805
Section #2	130.858	103.672	139.380	147.069
Section #3	131.479	80.108	165.289	177.740
Mean	124.362	97.007	144.256	137.538

Control embryo #4	RV		LV	
	CM thickness (µm)	TM thickness (µm)	CM thickness (µm)	TM thickness (µm)
Section #1	82.849	93.019	118.136	86.131
Section #2	69.683	75.859	131.617	76.034
Section #3	90.169	55.032	147.363	94.584
Mean	75,16666667	120,3333333	144,3333333	139

Control embryo #5	RV		LV	
	CM thickness (µm)	TM thickness (µm)	CM thickness (µm)	TM thickness (µm)
Section #1	112.440	83.278	156.679	75.862
Section #2	76.525	71.276	166.565	87.252
Section #3	135.594	72.434	161.922	94.072
Mean	75,16666667	120,3333333	144,3333333	139

**Mean ± S.E.M**	**RV**		**LV**	
	**CM thickness (µm)**	**TM thickness (µm)**	**CM thickness (µm)**	**TM thickness (µm)**
**Control**	**89.6 ± 9.8**	**117.0 ± 5.5**	**146.4 ± 2.1**	**142.2 ± 3.5**
Rbpj^{flox} ***;Tnnt2-Cre***	**84.7 ± 5.5**	**115.5 ± 7.6**	**143.4 ± 8.4**	**142.5 ± 5.7**

All measurements are in µm
RV = Right Ventricle
LV = Left ventricle
CM = Compact Myocardium
TM = Trabecular Myocardium

Mutant embryo #1	RV		LV	
	CM thickness (µm)	TM thickness (µm)	CM thickness (µm)	TM thickness (µm)
Section #1	99,3	103	138	123
Section #2	97,3	111	137	141
Section #3	98,7	85,5	147	126
Mean	98,43333333	99,83333333	140,6666667	130

Mutant embryo #2	RV		LV	
	CM thickness (µm)	TM thickness (µm)	CM thickness (µm)	TM thickness (µm)
Section #1	95,8	102	174	155
Section #2	92,3	77,9	157	181
Section #3	73,5	96,9	165	151
Mean	87,2	92,26666667	165,3333333	162,3333333

Mutant embryo #3	RV		LV	
	CM thickness (µm)	TM thickness (µm)	CM thickness (µm)	TM thickness (µm)
Section #1	82,7	119	131	144
Section #2	79,4	105	122	167
Section #3	89,5	110	149	152
Mean	83,86666667	111,3333333	134	154,3333333

Mutant embryo #4	RV		LV	
	CM thickness (µm)	TM thickness (µm)	CM thickness (µm)	TM thickness (µm)
Section #1	123,910	86,362	178,129	176,744
Section #2	59,751	131,950	197,205	178,827
Section #3	91,509	83,900	157,891	113,859
Mean	91,72333333	100,7373333	177,7416667	156,4766667

Mutant embryo #5	RV		LV	
	CM thickness (µm)	TM thickness (µm)	CM thickness (µm)	TM thickness (µm)
Section #1	93,211	128,699	139,244	105,177
Section #2	101,841	120,653	132,860	165,461
Section #3	107,941	111,322	136,610	136,599
Mean	100,9976667	120,2246667	136,238	135,7456667

Mutant embryo #6	RV		LV	
	CM thickness (µm)	TM thickness (µm)	CM thickness (µm)	TM thickness (µm)
Section #1	63,606	126,533	119,077	123,079
Section #2	61,006	82,254	108,223	115,876
Section #3	60,957	123,874	102,524	131,983
Mean	61,85633333	110,887	109,9413333	123,646

Mutant embryo #7	RV		LV	
	CM thickness (µm)	TM thickness (µm)	CM thickness (µm)	TM thickness (µm)
Section #1	60,475	81,883	108,800	143,423
Section #2	80,970	91,576	136,512	135,524
Section #3	65,202	97,749	175,231	125,600
Mean	68,88233333	90,40266667	140,181	134,849
