# Supplementary material for: Myocardial Notch1-Rbpj deletion does not affect NOTCH signaling, heart development or function
Source: PLoS One. 2018 Dec 31;13(12):e0203100. doi: 10.1371/journal.pone.0203100 (PMC6312338; doi:10.1371/journal.pone.0203100)
Supplement: S2 Table — (PDF) [file pone.0203100.s002.pdf]

Echocardiography analysis of 6 months old *Rbpjflox; Tnnt2-Cre*

	%EF	% FS	LV Mass	E/A	LVOT SV(uL)	LVOT CO(mL/	Tibia length(mm)	LV mass /Tibia	SV/TL (ul/mm)	CO/TL (ml/min*mm)
Control #1	53,415019	27,5079365	137,025441	1,34	90,165839	45,975	21	6,525021	4,293611381	2,189285714
Control #2	68,10847	37,424925	124,594812	1,75882353	75,1046865	34,05	19	6,557621684	3,952878237	1,792105263
Control #3	47,4283125	23,5714285	120,04203	2,07948718	67,3663555	32,625	22	5,456455909	3,062107068	1,482954545
Control #4	41,475669	20,212766	117,797533	2,33572977	50,0851025	24,475	21	5,60940631	2,385004881	1,16547619
Control #5	55,3276465	28,4965035	112,544499	2,0180965	46,8146175	21,615042	21	5,359261833	2,2292675	1,029287714
Control #6	45,937	22,6666665	123,106403	2,5170455	70,1264845	37,6034695	21	5,862209667	3,339356405	1,790641405
Control #7	62,5129735	33,9027775	135,202156	1,2709075	52,5920555	24,087859	21	6,438197905	2,504383595	1,147040905
Control #8	53,436038	27,4643875	122,781808	1,367486	56,5999405	22,661514	21	5,846752762	2,695235262	1,079119714
Control #9	69,4414385	38,1034485	83,955464	1,3133045	59,809072	28,6882975	21	3,997879238	2,848051048	1,366109405
Control #10	70,1183325	39,3851865	105,048071	1,806811	46,581865	21,2360545	21	5,002289071	2,218184048	1,01124069
Control #11	52,709359	26,811594	91,088906	0,697987	74,542181	31,89918	21	4,337566952	3,549627667	1,519008571
Control #12	47,889059	23,529412	166,466573	1,14375	118,379743	64,339245	21	7,926979667	5,637130619	3,063773571
Control #13	58	30	120,78	1,508065	71,792094	31,882996	22	5,49	3,263277	1,449227091
Mutant #1	65,3122485	35,5989805	101,13552	1,29566577	47,735976	25,13	18	5,618639972	2,651998667	1,396111111
Mutant #2	71,945379	39,9545455	88,345	2,31064421	46,8751745	16,275	19	4,649736842	2,467114447	0,856578947
Mutant #3	63,3920985	34,0409845	102,117613	1,55141326	52,627168	24,97	18	5,673200694	2,923731556	1,387222222
Mutant #4	66,7780605	36,589768	87,457061	1,14608037	42,942894	22,93	19	4,603003211	2,260152316	1,206842105
Mutant #5	62,2389465	33,3413075	59,5990415	1,66137649	41,7004515	17,535	19	3,136791658	2,194760605	0,922894737
Mutant #6	63,5046955	33,760684	77,780649	1,2533705	44,3237365	20,24	22	3,535484045	2,014715295	0,92
Mutant #7	63,1018915	33,3032785	66,062426	2,172517	58,3560915	20,658992	18	3,670134778	3,242005083	1,147721778
Mutant #8	57,8608755	29,7920895	106,631922	1,384425	60,4736295	28,46	19	5,612206421	3,182822605	1,497894737
Mutant #9	48,8524225	24,492908	106,795275	2,370484	59,369252	64,24	18	5,933070806	3,298291778	3,568888889
Mutant #10	47,0860945	23,4594595	156,539434	1,432898	38,8722265	20,67	19	8,238917553	2,045906658	1,087894737
Mutant #11	59,2244175	31,3861655	105,692083	1,2433255	92,48	33,775	20	5,284604125	4,624	1,68875
Mutant #12	57,3477123	29,4489328	71,3887468	1,565947	56,2384845	18,6930455	19	3,757302461	2,959920237	0,9838445
Mutant #13	59	31	81,41	2,57	40,77	20,96	18	4,522777778	2,265	1,164444444

Echocardiography analysis of 6 months old Notch1flox; Tnnt2-Cre

	%EF	% FS	LV Mass	E/A	LVOT SV(uL)	LVOT CO(mL/	Tibia length(mm)	LV mass /Tibia	SV/TL (ul/mm)	CO/TL (ml/min*mm)
Control #1	41,2656055	19,7574015	83,4392115	1,657316	36,6439385	17,6509815	20	4,171960575	1,832196925	0,882549075
Control #2	33,3404575	15,618779	94,6278535	1,7321855	35,3470925	17,81839	20	4,731392675	1,767354625	0,8909195
Control #3	41,0776535	19,8052705	94,391442	1,6463415	41,3775695	18,135478	20	4,7195721	2,068878475	0,9067739
Control #4	46,8899595	23,39375	103,639385	1,251019	37,2469725	16,2935655	20	5,18196925	1,862348625	0,814678275
Control #5	44,620753	21,6737335	89,8080975	2,046537	43,5528485	19,399757	20	4,490404875	2,177642425	0,96998785
Control #6	36,1329555	17,098506	104,896014	1,5040225	30,4570905	15,124633	20	5,244800675	1,522854525	0,75623165
Control #7	38,691616	18,3969465	97,2414635	2,292926	45,6840865	22,4657805	19	5,117971763	2,404425605	1,1824095
Control #8	44,4437115	21,7674295	104,09875	2,8076625	47,16951	21,064265	20	5,204937475	2,3584755	1,05321325
Mutant #1	44,2943585	21,5012145	70,0204985	1,6757185	35,545594	16,5006415	18	3,890027694	1,974755222	0,916702306
Mutant #2	45,4076255	21,983227	68,6994925	1,6403075	36,8120715	17,385162	18	3,816638472	2,045115083	0,965842333
Mutant #3	58,5364755	30,453104	66,63925	1,5578385	23,0854365	10,0854655	17	3,919955882	1,357966853	0,593262676
Mutant #4	53,6919005	27,144874	74,605915	1,855435	33,5750045	15,6796465	18	4,144773056	1,865278028	0,871091472
Mutant #5	44,1221965	21,1767205	71,935232	2,2398615	30,6452485	13,640253	19	3,786064842	1,612907816	0,717908053
Mutant #6	50,997804	25,362963	85,1954905	2,0444445	36,755613	18,312149	19	4,483973184	1,934505947	0,963797316
Mutant #7	44,880149	21,644881	63,769508	1,824048	32,5576565	17,2050855	18	3,542750444	1,808758694	0,955838083
Mutant #8	43,738704	21,089801	95,19105	1,549616	39,3139045	16,4674695	18	5,288391667	2,184105806	0,914859417

For each animal, two echocardiographies were performed with a two-weeks interval. Mean of these two measurements is represented for each parameter
