# Supplementary material for: Myocardial Notch1-Rbpj deletion does not affect NOTCH signaling, heart development or function
Source: PLoS One. 2018 Dec 31;13(12):e0203100. doi: 10.1371/journal.pone.0203100 (PMC6312338; doi:10.1371/journal.pone.0203100)
Supplement: S3 Table — (PDF) [file pone.0203100.s003.pdf]

Control #1	PR (ms)	QRS (ms)	RR (s)	QT (ms)	Corrected QT (ms)	BPM
Measurement #1	41,5	10,9	0,145	47	1234,28	412,37
Measurement #2	40	11,01	0,1375	46	1240,53	436,36
Measurement #3	41	11,4	0,141	47,5	1264,98	425,53
Mean	40,8333333	11,1033333	0,14116667	46,8333333	1246,596455	424,753333

Control #2	PR (ms)	QRS (ms)	RR (s)	QT (ms)	Corrected QT (ms)	BPM
Measurement #1	52	15,5	0,1525	53	1357,19	357,14
Measurement #2	52,5	14	0,1535	49	1250,67	352,94
Measurement #3	53	15	0,153	51	1303,84	366,97
Mean	52,5	14,8333333	0,153	51	1303,899661	359,016667

Control #3	PR (ms)	QRS (ms)	RR (s)	QT (ms)	Corrected QT (ms)	BPM
Measurement #1	46	14	0,121	47,5	1365,53	495,86
Measurement #2	45,5	15	0,121	52	1494,89	495,86
Measurement #3	46	14,5	0,12	49	1414,51	502,09
Mean	45,8333333	14,5	0,12066667	49,5	1424,977348	497,936667

Control #4	PR (ms)	QRS (ms)	RR (s)	QT (ms)	Corrected QT (ms)	BPM
Measurement #1	39	13,5	0,1155	37,5	1103,42	519,48
Measurement #2	39,5	14,5	0,116	39,5	1159,76	517,24
Measurement #3	40,5	13,5	0,117	35,5	1037,85	512,82
Mean	39,6666667	13,8333333	0,11616667	37,5	1100,343487	516,513333

Control #5	PR (ms)	QRS (ms)	RR (s)	QT (ms)	Corrected QT (ms)	BPM
Measurement #1	43,5	14,5	0,1615	48,5	1206,86	371,51
Measurement #2	44,5	13,5	0,1645	51	1257,44	364,74
Measurement #3	43	14,5	0,157	46,5	1173,55	382,16
Mean	43,6666667	14,1666667	0,161	48,6666667	1212,616685	372,803333

Control #6	PR (ms)	QRS (ms)	RR (s)	QT (ms)	Corrected QT (ms)	BPM
Measurement #1	47,5	15	0,153	51,5	1316,62	392,15
Measurement #2	46	15	0,156	48	1215,29	384,61
Measurement #3	46	15,5	0,153	48	1227,14	392,15
Mean	46,5	15,1666667	0,154	49,1666667	1253,018151	389,636667

Mutant #1	PR (ms)	QRS (ms)	RR (s)	QT (ms)	Tcorregido (m	BPM
Measurement #1	51,5	13,5	0,143	33	872,66	419,58
Measurement #2	50,5	13,5	0,14	34	908,69	428,5
Measurement #3	52	14	0,141	33,5	892,14	425,53
Mean	51,3333333	13,6666667	0,14133333	33,5	891,164844	424,536667

Mutant #2	PR (ms)	QRS (ms)	RR (s)	QT (ms)	Tcorregido (m	BPM
Measurement #1	48	16	0,155	47	1193,80	387,09
Measurement #2	48,5	15	0,157	51,5	1299,74	382,16
Measurement #3	49,5	15	0,1575	49,5	1247,28	380,95
Mean	48,6666667	15,3333333	0,1565	49,3333333	1246,94223	383,4

Mutant #3	PR (ms)	QRS (ms)	RR (s)	QT (ms)	Tcorregido (m	BPM
Measurement #1	49,5	15,5	0,1455	50	1310,81	412,37
Measurement #2	48,5	15,5	0,1475	46,5	1210,76	406,77
Measurement #3	48,5	13	0,162	46,5	1155,30	369,23
Mean	48,8333333	14,6666667	0,15166667	47,6666667	1225,62164	396,123333

Mutant #4	PR (ms)	QRS (ms)	RR (s)	QT (ms)	Tcorregido (m	BPM
Measurement #1	42	13	0,158	44,5	1119,52	379,74
Measurement #2	43	14	0,1585	48	1205,66	378,54
Measurement #3	41,5	14,5	0,161	47,5	1183,81	372,67
Mean	42,1666667	13,8333333	0,15916667	46,6666667	1169,66341	376,983333

Mutant #5	PR (ms)	QRS (ms)	RR (s)	QT (ms)	Tcorregido (m	BPM
Measurement #1	43	14	0,1795	50	1180,15	334,26
Measurement #2	43	14,5	0,1885	52,5	1209,22	318,3
Measurement #3	44	14,5	0,1795	47	1109,34	334,26
Mean	43,3333333	14,3333333	0,1825	49,8333333	1166,23642	328,94

Mutant #6	PR (ms)	QRS (ms)	RR (s)	QT (ms)	Tcorregido (m	BPM
Measurement #1	45,5	15	0,1545	46,5	1183,01	388,35
Measurement #2	47	17	0,156	44	1114,01	384,61
Measurement #3	46	17	0,1565	44,5	1124,87	383,38
Mean	46,1666667	16,3333333	0,15566667	45	1140,63181	385,446667

**Mean ± S.E.M**	**PR**	**QRS**	**QT**
**Control**	**44,8 ± 1,9**	**13,9 ± 0,6**	**47,1 ± 2,0**
Rbpj^{flox};Tnnt2-Cre	**46,7 ± 1,4**	**14,7 ± 0,4**	**43,3 ± 2,5**
