# Supplementary material for: Myocardial Notch1-Rbpj deletion does not affect NOTCH signaling, heart development or function
Source: PLoS One. 2018 Dec 31;13(12):e0203100. doi: 10.1371/journal.pone.0203100 (PMC6312338; doi:10.1371/journal.pone.0203100)
Supplement: S4 Table — (PDF) [file pone.0203100.s004.pdf]

| Control embryo #1 | RV | | LV | |
| --- | --- | --- | --- | --- |
| | CM thickness (µm) | TM thickness (µm) | CM thickness (µm) | TM thickness (µm) |
| Section #1 | 83,3 | 101 | 137 | 140 |
| Section #2 | 78,9 | 88,8 | 155 | 129 |
| Section #3 | 63,3 | 110 | 141 | 148 |
| Mean | 75,16666667 | 99,93333333 | 144,3333333 | 139 |

| Control embryo #2 | RV | | LV | |
| --- | --- | --- | --- | --- |
| | CM thickness (µm) | TM thickness (µm) | CM thickness (µm) | TM thickness (µm) |
| Section #1 | 101 | 72,1 | 161 | 161 |
| Section #2 | 101 | 73,3 | 153 | 151 |
| Section #3 | 92,5 | 111 | 150 | 157 |
| Mean | 98,16666667 | 85,46666667 | 154,6666667 | 156,3333333 |

| Control embryo #3 | RV | | LV | |
| --- | --- | --- | --- | --- |
| | CM thickness (µm) | TM thickness (µm) | CM thickness (µm) | TM thickness (µm) |
| Section #1 | 110.750 | 107.242 | 128.099 | 87.805 |
| Section #2 | 130.858 | 103.672 | 139.380 | 147.069 |
| Section #3 | 131.479 | 80.108 | 165.289 | 177.740 |
| Mean | 124.362 | 97.007 | 144.256 | 137.538 |

| Control embryo #4 | RV | | LV | |
| --- | --- | --- | --- | --- |
| | CM thickness (µm) | TM thickness (µm) | CM thickness (µm) | TM thickness (µm) |
| Section #1 | 82.849 | 93.019 | 118.136 | 86.131 |
| Section #2 | 69.683 | 75.859 | 131.617 | 76.034 |
| Section #3 | 90.169 | 55.032 | 147.363 | 94.584 |
| Mean | 75,16666667 | 120,3333333 | 144,3333333 | 139 |

| Control embryo #5 | RV | | LV | |
| --- | --- | --- | --- | --- |
| | CM thickness (µm) | TM thickness (µm) | CM thickness (µm) | TM thickness (µm) |
| Section #1 | 112.440 | 83.278 | 156.679 | 75.862 |
| Section #2 | 76.525 | 71.276 | 166.565 | 87.252 |
| Section #3 | 135.594 | 72.434 | 161.922 | 94.072 |
| Mean | 108.186 | 75.663 | 161.722 | 85.729 |

| Control embryo #6 | RV | | LV | |
| --- | --- | --- | --- | --- |
| | CM thickness (µm) | TM thickness (µm) | CM thickness (µm) | TM thickness (µm) |
| Section #1 | 67,7 | 111 | 92,5 | 139 |
| Section #2 | 45 | 113 | 120 | 135 |
| Section #3 | 62,5 | 115 | 167 | 175 |
| Mean | 58,4 | 113 | 126,5 | 149,6666667 |

| **Mean ± S.E.M** | **RV** | | **LV** | |
| --- | --- | --- | --- | --- |
| | **CM thickness (µm)** | **TM thickness (µm)** | **CM thickness (µm)** | **TM thickness (µm)** |
| **Control** | **86.2 ±11.4** | **116.2 ±55.5** | **142.8 ±4.5** | **144.3 ±3.7** |
| ***Rbpj$^{flox}$;Nkx2.5-Cre*** | **99.0 ±19.0** | **131.9 ±6.5** | **167.0 ±16.8** | **151.1 ±12.2** |

| Mutant embryo #1 | RV | | LV | |
| --- | --- | --- | --- | --- |
| | CM thickness (µm) | TM thickness (µm) | CM thickness (µm) | TM thickness (µm) |
| Section #1 | 150 | 164 | 160 | 131 |
| Section #2 | 134 | 134 | 182 | 186 |
| Section #3 | 125 | 125 | 180 | 148 |
| Mean | 136,3333333 | 141 | 174 | 155 |

| Mutant embryo #2 | RV | | LV | |
| --- | --- | --- | --- | --- |
| | CM thickness (µm) | TM thickness (µm) | CM thickness (µm) | TM thickness (µm) |
| Section #1 | 72,7 | 121 | 134 | 173 |
| Section #2 | 72,6 | 129 | 146 | 136 |
| Section #3 | 77,1 | 156 | 125 | 201 |
| Mean | 74,13333333 | 135,3333333 | 135 | 170 |

| Mutant embryo #3 | RV | | LV | |
| --- | --- | --- | --- | --- |
| | CM thickness (µm) | TM thickness (µm) | CM thickness (µm) | TM thickness (µm) |
| Section #1 | 78,3 | 193 | 157 | 125 |
| Section #2 | 90,9 | 46 | 189 | 130 |
| Section #3 | 90,3 | 119 | 230 | 130 |
| Mean | 86,5 | 119,3333333 | 192 | 128,3333333 |

All measurements are in µm
RV = Right Ventricle

LV = Left ventricle
CM = Compact Myocardium
TM = Trabecular Myocardium
