# Supplementary material for: Myocardial Notch1-Rbpj deletion does not affect NOTCH signaling, heart development or function
Source: PLoS One. 2018 Dec 31;13(12):e0203100. doi: 10.1371/journal.pone.0203100 (PMC6312338; doi:10.1371/journal.pone.0203100)
Supplement: S5 Table — (PDF) [file pone.0203100.s005.pdf]

Strain	Genotyping primers
CBF:H2B-Venus	F 5´ GCTGATTAATCGAGATCTGGTGTAAACAC 3´
	R 5´ GCGAGCTAGCCAGCTTTTTGCAAAAGCCTAG 3´
Rbpj ^{flox}	F 5´ ACC AGA ATC TGT TTG TTA TTT GCA TTA CTG 3´
	R 5´ ATG TAC ATT TTG TAC TCA CAG AGA TGG ATG 3´
Tnnt2-Cre	F 5´ TAC TCA AGA ACT ACG GGC TGC 3´
	R 5´ GCA CTC CAG CTT GGT TCC CGA 3´
Nkx2.5-Cre	F 5´ GCG CAC TCA CTT TAA TGG GAA GAG 3´
	R 5´ GCC CTG TCC CTC AGA TTT CAC ACC 3´
Notch1 ^{flox}	F 5' CTGAGGCCTAGAGCCTTGAA 3'
	R 5' TGTGGGACCCAGAAGTTAGG 3'
ROSA $^{mT/mG}$	F 5´ CTC TGC TGC CTC CTG GCT TCT 3´
	R 5´ TCA ATG GGC GGG GGT CGT T 3´

Gene	qRT-PCR primers
Hif1α	F 5´ GGAGCCTTAACCTGTCTGCC 3´
	R 5´ GCAGTCTGCATGCTAAATCGG 3´
Vegfa	F 5´ TAGAGTACATCTTCAAGCCG 3´
	R 5´ TCTTTCTTTGGTCTGCATTC 3´
Rbpj	F 5´ ACAGTGCTCATTCTTCAT3´
	R 5´ CATCCATCTGGTTCCATT 3´
Gapdh	F 5´ AACTTTGGCATTGTGGAAGG 3´
	R 5´ ACACATTGGGGGTAGGAACA 3´
